# A Cascaded Neural Network for Staging in Non-Small Cell Lung Cancer Using Pre-Treatment CT

**DOI:** 10.3390/diagnostics11061047

**Published:** 2021-06-07

**Authors:** Jieun Choi, Hwan-ho Cho, Junmo Kwon, Ho Yun Lee, Hyunjin Park

**Affiliations:** 1 Department of Artificial Intelligence, Sungkyunkwan University, Suwon 16419, Korea; line7220@g.skku.edu; 2 Department of Electrical and Computer Engineering, Sungkyunkwan University, Suwon 16419, Korea; guraud0810@skku.edu (H.-h.C.); skenfn1231@skku.edu (J.K.); 3 Center for Neuroscience Imaging Research, Institute for Basic Science, Suwon 16419, Korea; 4 Department of Radiology and Center for Imaging Science, Samsung Medical Center, Sungkyunkwan University School of Medicine, Seoul 06351, Korea; hoyunlee96@gmail.com; 5 Department of Health Sciences and Technology, Samsung Advanced Institute for Health Sciences & Technology (SAIHST), Sungkyunkwan University, Seoul 06351, Korea; 6 School of Electronic and Electrical Engineering, Sungkyunkwan University, Suwon 16419, Korea

**Keywords:** non-small cell lung cancer, computed tomography, deep learning, convolutional neural network, autoencoder

## Abstract

Background and aim: Tumor staging in non-small cell lung cancer (NSCLC) is important for treatment and prognosis. Staging involves expert interpretation of imaging, which we aim to automate with deep learning (DL). We proposed a cascaded DL method comprised of two steps to classification between early- and advanced-stage NSCLC using pretreatment computed tomography. Methods: We developed and tested a DL model to classify between early- and advanced-stage using training (*n* = 90), validation (*n* = 8), and two test (*n* = 37, *n* = 26) cohorts obtained from the public domain. The first step adopted an autoencoder network to compress the imaging data into latent variables and the second step used the latent variable to classify the stages using the convolutional neural network (CNN). Other DL and machine learning-based approaches were compared. Results: Our model was tested in two test cohorts of CPTAC and TCGA. In CPTAC, our model achieved accuracy of 0.8649, sensitivity of 0.8000, specificity of 0.9412, and area under the curve (AUC) of 0.8206 compared to other approaches (AUC 0.6824–0.7206) for classifying between early- and advanced-stages. In TCGA, our model achieved accuracy of 0.8077, sensitivity of 0.7692, specificity of 0.8462, and AUC of 0.8343. Conclusion: Our cascaded DL model for classification NSCLC patients into early-stage and advanced-stage showed promising results and could help future NSCLC research.

## 1. Introduction

Non-small cell lung cancer (NSCLC) accounts for 85% of lung cancers and proper staging provides critical information for treatment and prognosis [1]. Staging tumors depends on image interpretations performed by experts [2]. The current staging of NSCLC is based on the size and extent of the tumor, the degree of spread to lymph nodes, and the degree of metastasis [3]. Since improving survival in advanced patients with stage Ⅱ-Ⅳ is difficult and treatment methods should be tailored to the progress of the disease, accurate staging of early-stage patients is crucial [4,5].

Radiomics is an approach where features related to staging are available [6]. Imaging data are transformed into mathematically defined high-dimensional features focusing on tumor appearance and intra-tumoral heterogeneity and researchers can mine features related to diagnosis and prognosis [7]. Palumbo et al. [8] used shape and texture features from PET/CT to classify solitary pulmonary nodule into malignant and benign and showed better performance than using standard imaging features alone. Features adopted in radiomics are considered as handcrafted features because they are based on the experts’ mathematical knowledge [9]. There are hundreds and sometimes thousands of features in radiomics and thus the features are likely to include information needed in staging [6]. Even if there are many radiomics features, we could still be missing uncovered abstract information from imaging data that is not properly modeled according to current knowledge [10].

Recently, a subdiscipline of machine learning known as deep learning (DL) has emerged and made a significant impact in medical image analysis, capitalizing on the advances of computing hardware, algorithms, and big data [10]. The neural networks of DL extract features in a data-driven fashion and thus could uncover potentially missing abstract radiomics information using handcrafted features. Noting this potential, Bianconi et al. [11] applied deep learning methods for semi-automated segmentation of pulmonary nodules on CT and showed that deep learning methods outperformed conventional methods such as edge detection. Paing et al. [12] applied back propagation network and machine learning methods to classify T-stage of lung tumors. Nibali et al. [13] extracted lung nodule patches from coronal, sagittal, and axial views and used three separate DL networks to distinguish between benign and malignant nodules in lung cancer. Ciompi et al. [14] used an ensemble classifier to classify pulmonary peri-fissural nodules. Jiang et al. [15] proposed multi-resolution residual networks to perform lung tumor segmentation.

**Figure 1:**
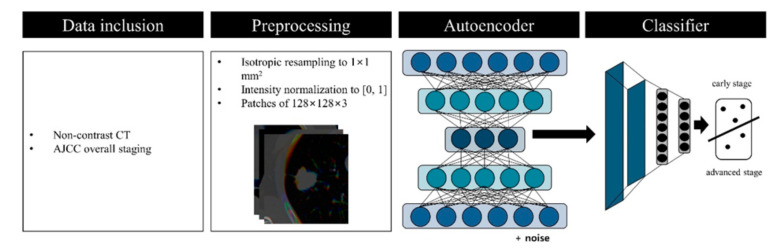
Overall workflow.

In this thesis, we proposed a serial DL method to classify between early- and advanced-stage NSCLC using pretreatment computed tomography (CT). Our method was trained and tested for overall pathological staging information from six publicly available datasets. The first step of our method contained an autoencoder to compress the imaging data into latent variables. The second step used the latent variable to classify the stages using the convolutional neural network (CNN). The overall workflow of this study is shown in Figure 1. Our main contribution was to adapt a well-established DL methodology to solve an important clinical problem.

## 2. Materials and Methods

### 2.1. Patient Cohorts and Imaging Data

We obtained six NSCLC cohorts with pathological staging information from the public domain of the cancer imaging archive (TCIA). This study was a retrospective analysis of anonymized data and institutional review board (IRB) approval was obtained at Sungkyunkwan University. All data were obtained with informed written consent. The cohorts were NSCLC-Radio-genomics [16,17,18,19], NSCLC-Radiomics-Genomics [19,20,21], CPTAC-LUAD [19,22], CPTAC-LSCC [19,23], TCGA-LUAD [19,24], and TCGA-LUSC [19,25] cohorts. The CPTAC-LUAD and TCGA-LUAD contained lung adenocarcinoma and the CPTAC-LSCC and TCGA-LUSC contained lung squamous cell carcinoma. The first two cohorts were combined and used as training and validation sets. The CPTAC-LUAD and CPTAC-LSCC cohorts were combined and used as the first test set. The TCGA-LUAD and TCGA-LUSC cohorts were combined and used as the second test set. Some patients had both contrast-enhanced and non-contrast CT, while some patients had only one. We included patients with non-contrast CT that led a total of 65 in NSCLC-Radio-genomics, 33 in NSCLC-Radiomics-Genomics, 17 in CPTAC-LUAD, 20 in CPTAC-LSCC, 13 in TCGA-LUAD, and 13 in TCGA-LUSC cases. The cases were further grouped into training (*n* = 90), validation (*n* = 8), CPTAC-test cohorts (*n* = 37) and TCGA-test cohort (*n* = 26). Details regarding patient information are given in Table 1. The six cohorts had non-contrast CT imaging performed with various scanners, obtained with the following parameters: detector collimation 0.3184 to 1.3672 mm; reconstruction interval 0.5 to 5 mm. The most typical CT imaging setting was the 0.625 mm detector collimation and 1.34 mm reconstruction interval. Some cohorts, NSCLC-Radio-genomics and NSCLC-Radiomics-Genomics, did not provide the overall pathological stage, but provided TNM staging information. TNM represents the size and extent of the main tumor, the spread to nearby lymph nodes, and the metastasis to distant sites, respectively. Thus, we computed the overall stage using the available TNM stage information provided by the open database according to the American Joint Committee on Cancer staging manual (7th version) [3]. The stages were binarized to early-stage (stage Ⅰ) and advanced-stage (stages Ⅱ-Ⅳ).

The NSCLC-Radio-genomics and NSCLC-Radiomics-Genomics cohorts were combined into one set and we randomly split them into training cohort (*n* = 90) and validation cohort (*n* = 8) keeping the relative frequency of early- and advanced-stage (i.e., 0.63 and 0.37) similar between cohorts. The validation cohort was used to tune the hyperparameters of the two networks. We combined CPTAC-LUAD and CPTAC-LSCC cohorts to form the first test cohort (CPTAC-test, *n* = 37) and combined TCGA-LUAD and TCGA-LUSC to form the second test cohort (TCGA-test, *n* = 26). The datasets were assigned based on the data collection institutions.

### 2.2. Data Preprocessing

We focused on primary tumors. For cohorts with tumor region of interest (ROI) (i.e., NSCLC-Radio-genomics and NSCLC-Radiomics-Genomics), the center of the tumor was computed from the centroid of the ROI and for cohorts without ROI (i.e., CPTAC-LUAD, CPTAC-LSCC, TCGA-LUAD, and TCGA-LUSC), the center of the tumor was manually specified by a thoracic radiologist (H.Y.L with 15 years of experience). Because the imaging data were collected from multi-sites, we resampled the CT images to isotropic 1 mm resolution to make a fair comparison among different cohorts. CT images were resampled by b-spline interpolation methods and ROIs were resampled by the nearest neighbor method. Each CT image was cropped into one patch with size 128 × 128 × 3 whose center slice showed the largest spatial extent of the tumor in 2D. The in-plane extent of 128 mm was chosen to accommodate the largest tumor. CT intensities were linearly normalized to lie between 0 and 1.

### 2.3. Autoencoder Network to Extract Latent Variables

**Figure 2:**
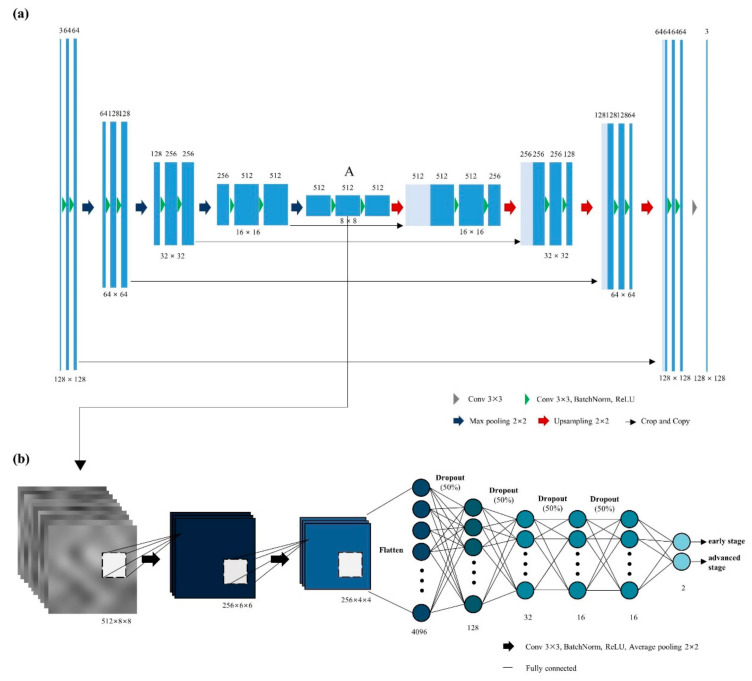
Model architectures. (**a**) U-net autoencoder for reconstructing CT images. (**b**) Classification networks for classifying early- and advanced-stage utilizing latent variable (noted with A) from (**a**) as input.

U-net is a deep neural network that works well in many medical imaging analysis tasks such as segmentation, registration, and reconstruction [26]. Here we used it to extract latent variables of the input images while the network tried to reconstruct the original image. Our U-net architecture consisted of contracting and expanding paths with several blocks. Each block had two CNN layers with kernel size 3 × 3, batch normalization, and rectified linear unit (ReLU). In the contracting paths, the blocks were connected with max pooling layer with stride 2 for down-sampling. Up-sampling layer with bilinear interpolation was used for connections between blocks in the expanding paths. A total of 23 convolutional layers were employed in our U-net. Figure 2 shows the detail of the U-net autoencoder architecture. We extracted latent variables of size 8 × 8 × 512 from the last layer of the contracting path before ReLU. To train the autoencoder model to reconstruct the original images, we used a fixed set of input images with Gaussian noise (mean = 0 and standard deviation = 0.1) added. The mean square error (MSE) loss and stochastic gradient descent (SGD) with a learning rate of 1 × 10^−2^ with a batch size of 16, and 0.9 momentum were used.

### 2.4. Classification Network for Staging

To classify the patients into early- or advanced- stages, we employed an architecture based on CNN followed by a fully connected (FC) layer using latent variables obtained from the autoencoder as inputs. Figure 2 shows the architecture of the classification network. The network consisted of two CNN layers of 256 filters with a kernel size of 3 × 3 and padding size 1 for each direction. Between the CNN layers, batch normalization, ReLU, and 3 × 3 average pooling were adopted. CNN layers were followed by five FC layers with 128, 32, 16, 16, and 2 nodes, respectively. Dropout (ratio 0.5) was applied between all layers. Final prediction probabilities were calculated after the fifth FC layer by softmax. The cross-entropy loss and gradient-based stochastic optimizer Adam with a learning rate of 1 × 10^−6^ and a batch size of 16 was used.

### 2.5. Comparison with Other Models

We compared our model in four aspects. First, we compared with autoencoder models that compressed the input images further with higher compression ratios. Our U-net network described in Section 2.3 used 3, 64, 128, 256, 512, and 512 channels to compress the features along the blocks. To make more compressed latent variables, we designed two U-net networks that share the same backbone as our proposed model except for the number of channels in each block. In the first compared model, the number of channels was 3, 32, 64, 128, 256, and 256 in each block and resulted in 8 × 8 × 256 latent variables. In the second compared model, the number of channels was with 3, 16, 32, 64, 128, and 128 in each block and resulted in 8 × 8 × 128 latent variables. The two models were referred to as U-net (256) and U-net (128) models based on the size of the compressed feature maps. The SGD with a learning rate of 1 × 10^−2^, a batch size of 16, and 0.9 momentum were used for both models.

Second, we compared ours with a basic CNN-based network in terms of image reconstruction. In the basic CNN reconstruction model, each block in U-net was replaced with a single CNN layer without residual connection. The basic CNN reconstruction model had contracting and extracting paths as the U-net based autoencoder. The basic CNN reconstruction model was designed to have the same down sample steps as our autoencoder so we could extract the equal-sized latent variables after the contracting path. The contracting path consisted of five layers with 3 × 3 CNN layer and 2 × 2 max pooling layer with stride 2 for down-sampling. The expanding path repeated the layers in the contracting path except that the max-pooling layer was replaced with an up-sampling layer. The SGD with a learning rate of 1 × 10^−1^, a batch size of 16, and 0.9 momentum were used.

Third, we compared our classification networks with other approaches. Our classification networks were compared with support vector machine (SVM) and random forest methods. We also compared ours with a network where latent variables were extracted from the basic CNN reconstruction model. The latent variables of the basic CNN reconstruction model were extracted at the end of the contracting path whose size was 8×8×512 to keep the size of the latent variable the same as ours.

Finally, we compared ours with a fine-tuned single-stage model of pre-trained ResNet50 using 128 × 128 image patches as input to classify between the early- and advanced stages.

### 2.6. Statistical Analysis

We used ANOVA to compare continuous-valued information and the Chi-square test to compare categorized information in the demographics. Classification performance was measured with accuracy, sensitivity, specificity, and area under the curve (AUC). All statistical analyses were performed with the Statistics Toolbox of “scipy” and “statsmodels” in Python.

### 2.7. Training Setup

Pytorch (version 1.4.0) was used to build our neural networks. Our U-net autoencoder was trained for 300 epochs and the classification network was trained for 500 epochs. For each network, we utilized early stopping where the validation loss was the minimum. It took 6 min 40 s for training our U-net autoencoder and took 44 s for training classification network using NVIDIA GeForce RTX 2070 SUPER graphics card. Our code is available at GitHub: https://github.com/Jieun1203/Classifying-Non-small-cell-lung-cancer-stage.

## 3. Results

### 3.1. Clinical Characteristics of Cohorts

Demographic information of study cohorts is attached in Table 1. No difference was found among NSCLC-Radio-genomics, NSCLC-Radiomics-Genomics, CPTAC-test, and TCGA-test cohorts in either age (*p* = 0.9023) and sex (*p* = 0.6943). The distribution of staging (early- and advanced-stage) in training, validation, and test cohorts is described in Table 2. A significant difference was not observed in staging (*p* = 0.5777).

### 3.2. Reconstructing Images Using U-Net Autoencoder

**Figure 3:**
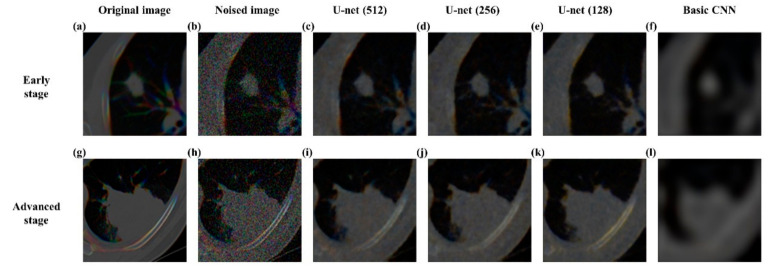
Representative reconstruction results of our U-net autoencoder, U-net (256), U-net (128), and basic CNN reconstruction model. (**a**) Original image of NSCLC patients in early-stage (**b**) noise added images of NSCLC patients in early-stage (**c**) reconstructed images of the proposed U-net (512) autoencoder in early-stage (**d**) reconstructed images of U-net autoencoder using latent variables with 256 channels in early-stage (**e**) reconstructed images of U-net autoencoder using latent variables with 128 channels in early-stage (**f**) reconstructed images of the basic CNN reconstruction in early-stage (**g**) original image of NSCLC patients in advanced-stage (**h**) noise added images of NSCLC patients in advanced-stage (**i**) reconstructed images of the proposed U-net autoencoder (512) in advanced-stage (**j**) reconstructed images of U-net autoencoder using latent variables with 256 channels in advanced-stage (**k**) reconstructed images of U-net autoencoder using latent variables with 128 channels in advanced-stage (**l**) reconstructed images of the basic CNN reconstruction in advanced-stage.

Our U-net autoencoder performed well in reconstructing the original image with MSE as 0.0007 in the test cohort. In comparison, the U-net (256) showed MSE 0.0008, the U-net (128) performed reconstruction with MSE 0.0009, and the basic CNN reconstruction model resulted in an MSE of 0.0019. Figure 3 shows qualitative differences between our U-net autoencoder and the basic CNN reconstruction model in reconstructing original images both in early-stage and advanced-stage cases.

### 3.3. Classification of Early and Advanced Stages

Our model was compared with various approaches as shown in Table 3. Our model achieved accuracy of 0.8649, sensitivity of 0.80, specificity of 0.9412, and AUC of 0.8206 in the CPTAC-test cohort showing higher performance in most of the performance criteria. The approach that combined U-net denoising autoencoder with SVM achieved AUC of 0.7176, while the approach combining U-net denoising autoencoder with random forest achieved AUC of 0.7206. Using the basic CNN reconstruction model with our classification networks showed AUC of 0.6824. We also compared our results with previous studies.

**Figure 4:**
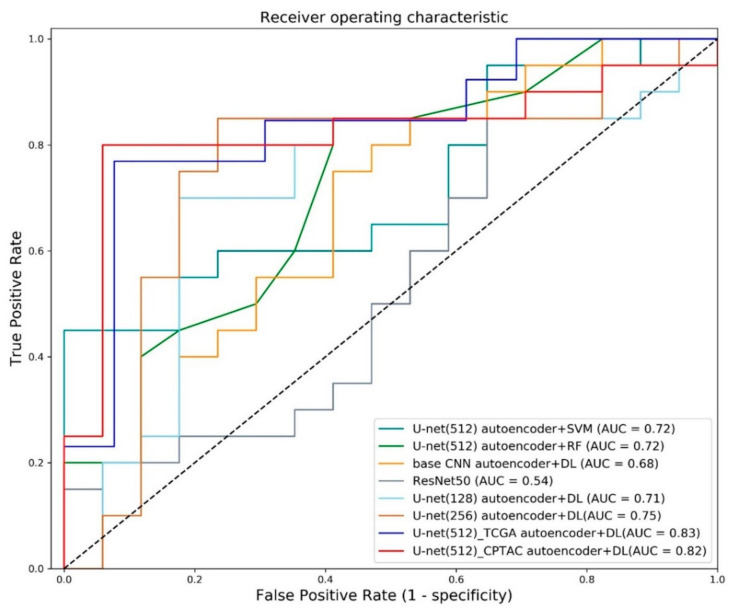
Receiver operating characteristic curve. ROC curves of various models. Curves correspond to SVM with latent variable of U-net (512) autoencoder, random forest with latent variable of U-net (512) autoencoder, deep learning classifier with latent variable of basic CNN reconstruction, single-stage ResNet50, deep learning classifier with latent variable from U-net (128), deep learning classifier with latent variable from U-net (256) and deep learning classifier with latent variable of U-net (512) autoencoder. The AUC of our methods is higher than others in both test cohorts.

To distinguish between early- and advanced-stages. Sun et al. (2018) [27] used a deep restricted Boltzmann machine to predict each NSCLC staging (i.e., stages Ⅰ to Ⅲb). The mean AUC for predicting stage Ⅰ, stage Ⅱ, stage Ⅲa, stage Ⅲb, was 0.69. The AUC for predicting stage Ⅰ, the same task as ours, was 0.67. Utilizing the Resnet50 showed an AUC of 0.5441. Using more compressed latent variable from U-net (128) achieved AUC of 0.7088 and using U-net (256) led to showed AUC of 0.7529. We also tested our model in the TCGA-test cohort, which led to an AUC of 0.8343. Figure 4 shows the receiver operating characteristic (ROC) curve plots for various approaches.

Some researchers dichotomized early- and advanced-stages using stage III as the cutoff (i.e., stage Ⅰ-Ⅱ vs. stage Ⅲ-Ⅳ) [28]. They extracted radiomics features from CT and selected important features that were used in random forest to classify between early-stage (stage Ⅰ-Ⅱ) and advanced-stage (stage Ⅲ-Ⅳ) in TCGA-LUAD and TCGA-LUSC. For the TCGA-LUAD portion, the study showed performances of 0.2857 (accuracy), 0 (sensitivity), 1 (specificity), and 0.80 (AUC). For the TCGA-LUSC portion, these were 0.6296 (accuracy), 0.75 (sensitivity), 0.4545 (specificity), and 0.68 (AUC). These performance numbers were obtained from literature and thus were not directly comparable to our results because of cutoff to dichotomize stages, and inclusion criterion.

### 3.4. Possible Confirmation Using Activation Maps

**Figure 5:**
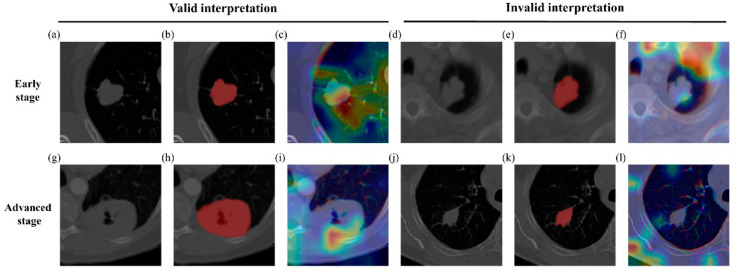
Grad-CAM results. Grad-CAM of classification results. Red represents high contribution and blue stands for low contribution for classification. The first row, (**a**–**f**), has cases of early-stage samples and the second row, (**g**–**l**) has cases of advanced-stage samples. The first column, (**a**,**g**), has input CT images for valid interpretations, while the fourth column, (**d**,**j**), has input CT images for invalid interpretations. The second column, (**b**,**h**), shows the ground truth tumor areas for valid interpretations. The fifth column, (**e**,**k**), shows the ground truth tumor areas for invalid interpretations. The third column, (**c**,**i**), has activation maps from (**a**,**g**). The last column, (**f**,**l**), has activation maps from (**d**,**j**).

We adopted gradient-weighted class activation mapping (grad-CAM) [29] to provide potential confirmations of our approach. The grad-CAM maps were computed from the second CNN layers in the classification network. As shown in Figure 5, the activations were mostly focused on the tumor area for successful classification cases. For unsuccessful cases, the activation maps focused on other areas such as bone and non-lung regions rather than primary tumor and led to incorrect classifications.

## 4. Discussion

We proposed a serial deep learning network model to classify NSCLC patients into early-stage and advanced-stage using pretreatment CT. Our U-net autoencoder used the input of noisy CT images to reconstruct CT images robustly and obtained latent variables that were robust and compact representations of the CT. The ensuing classification network of CNN followed by FC layers was able to perform binary classification well between early-stage and advanced-stage (AUC of 0.8206 and 0.8343) outperforming existing studies.

Our approach has the advantage of extracting compact data-driven features using the autoencoder. Our autoencoder-derived features could be novel features not described by existing handcrafted features, which might account for the improved performance. Another benefit is reduced human intervention. Previous methods, especially radiomics approaches, require tumor ROIs that require expert annotation [30], while our approach only requires the tumor center to be specified, which takes less effort.

Our autoencoder model adopted a 2.5D model, which considered three consecutive axial patches centered at the tumor centroid, not the full 3D model where the full extent of the lung parenchyma spanning was considered. A full 3D model is likely to extract more comprehensive information from the whole lung besides the tumor, but it comes at a cost of high computational resources and an increased sample size. 3D datasets would have different sizes, so we might need to fix the size for proper training. Since our model is a 2.5D model with only a few patches to consider, our model has a lighter computational load with reduced sample size requirements.

Our latent variables were extracted from the U-net autoencoder. There was a significant performance difference (AUC 0.82 vs. 0.68) between using latent variables from U-net autoencoder and the basic CNN reconstruction model. This showed that our autoencoder was able to produce compact and robust features related to staging. In related work, Cui et al. (2019) [31] extracted latent variables from the variational autoencoder (VAE) and successfully applied them for predicting radiation pneumonitis. VAE has the advantage of regularizing the latent variable for better generalization potential, which we plan to adopt in future studies.

Our method shows better performance than radiomics, conventional machine learning methods (SVM and random forest), and a single-stage ResNet50 as shown in Table 3. Radiomics tends to rely on handcraft features while ours can extract abstract latent variables tailored to the data and hence could lead to better performance [8]. The latent variables were subjected to the deep neural network, SVM, and random forest classifiers and our approach fared the best due to the increased complexity of many layers in the deep neural network. The single-stage ResNet50 has more complexity than ours and could suffer from overfitting the training data, especially when the training sample size is not big enough [32].

Visualization of the activation maps showed that correct classification might have occurred when the attention was focused on the tumor area and more importantly on the margin of the tumor (Figure 5). The region of tumor margin is an important region where dynamic interaction occurs between tumor cells and the surrounding microenvironment [33]. Our activation confirmed that this important tumor margin might have played a role in the decision making of our algorithm.

The cut-off to separate early- and advanced-stage in NSCLC could be either stage Ⅰ or Ⅱ. We grouped stage Ⅰ as early-stage and other stages as advanced-stage following an existing study [4]. Others assigned stage Ⅰ and Ⅱ as early-stage and other stages as advanced-stage [34]. We chose the former approach because it led to having a more balanced number of samples in early- and advanced-stage groups.

Our datasets were entirely from the public domain that led to heterogeneity in image acquisition. This was partly mitigated by resampling the imaging data to 1 mm isotropic resolution. Still, there is heterogeneity within and across training, validation, and test cohorts. However, many datasets from the clinic are heterogeneous in image acquisition and thus our developed model might better reflect the clinical reality.

Our model focused on image patches with tumor. However, it is possible to feed the whole slice image instead of the tumor patch to the neural network, but with that approach less important information of background and normal tissue is fed to the network. With limited samples, we needed to focus on the important tumor region. We built another model where resized whole slices were fed and observed that performed worse than our main model. The whole slice model showed accuracy of 0.7027, sensitivity of 1.0, specificity of 0.2353, and AUC of 0.5647.

Our study has some limitations. First, we adopted a 2.5D model to lessen the computation burden. However, a full 3D model might extract more powerful latent variables for staging. This needs to be explored in future studies using a larger cohort. Second, our method requires the center of the tumor to be specified. This was possible when ROI information was available. For data without the ROI, a separate method to segment the tumor ROI is necessary. There are studies showing high performance for segmentation [35,36,37]. These have the potential to detect tumor centers well. We hope to use this technology combined with increased samples to construct an end-to-end automatic network for tumor grading in the future. In a similar vein, we plan to perform a three-way classification of normal, early-stage, and advance-stage using DL approaches. Third, DL models tend to scale well in performance as sample sizes are increased. Although we showed generalizable performance in two independent test cohorts, having more samples could lead to better models. Future studies need to apply more complex DL models using larger cohorts to improve performance. Fourth, our data had enough samples in dichotomous classification into early- vs. advanced-stages, but we had very few samples in stages Ⅲ and Ⅳ due to the limitation of the public database. With enough samples in each stage, further studies could be designed to fully model the distribution of all four stages. Lastly, we dichotomized the stages without considering whether the patient received surgery due to the limitation of open datasets. In the future, we look forward to incorporating surgical treatment when dichotomizing stages.

## 5. Conclusions

In this study, we proposed a cascaded neural network for automated staging in NSCLS using pretreatment CT. Our proposed method performed the binary classification between early-stage and advanced-stage well (AUC of 0.8206 and 0.8343) outperforming existing studies. The results of our study might be useful in future DL studies of NSCLC.

## Figures and Tables

**Table 1:** Patient information for various cohorts.

	Training and Validation		CPTAC-Test		TCGA-Test		
	NSCLC-Radio-Genomics	NSCLC-Radiomics-Genomics	CPTAC- LUAD	CPTAC- LSCC	TCGA- LUAD	TCGA- LUSC	*p*-Value
*n*	65	33	17	20	13	13	
Age mean (STD)	68.97 (9.1532)	N/A	68.47 (6.30)	68.05 (6.36)	68.08 (10.56)	68 (11.53)	0.9023
Sex							0.6943
Male	49	25	10	13	8	9	
Female	16	8	7	7	5	4	
N stage							
N0	54	24	15	16	10	9	
N1	4	7	2	4	-	3	
N2	7	2	-	-	3	1	
Early stage							
Stage Ⅰ	45	17	8	12	8	5	
Advanced-stage							
Stage Ⅱ	9	13	11	4	1	6	
Stage Ⅲ	9	2	1	1	3	2	
Stage Ⅳ	2	1	-	-	1	-	

**Table 2:** Patients information for training, validation, and test cohorts.

	Training	Validation	CPTAC-Test	TCGA-Test	*p*-Value
Stage					0.5777
Early-	57	5	20	13	
Advanced-	33	3	17	13	
Total	90	8	37	26	

**Table 3:** Performance comparison of various models.

	Accuracy	Sensitivity	Specificity	AUC
U-net autoencoder + SVM	0.62	0.80	0.41	0.72
U-net autoencoder + random forest	0.62	1.0	0.18	0.72
Basic CNN reconstruction + DL	0.57	0.45	0.71	0.68
Sun et al. (2018) [25]	-	-	-	0.67
Single-stage ResNet50	0.62	0.85	0.35	0.54
U-net (128) autoencoder + DL	0.76	0.70	0.82	0.71
U-net (256) autoencoder + DL	0.78	0.85	0.71	0.75
Proposed U-net (512) autoencoder + DL (TCGA)	0.81	0.77	0.85	**0.83**
Proposed U-net (512) autoencoder + DL (CPTAC)	**0.86**	0.80	**0.94**	0.82

Note. The best performance of each evaluation criteria is shown in bold.

## Data Availability

The data presented in this study are openly available in *TCIA* http://doi.org/10.7937/K9/TCIA.2017.7hs46erv (Accessed on 15 July 2020) [16], *TCIA* https://doi.org/10.7937/K9/TCIA.2015.L4FRET6Z (Accessed on 15 July 2020) [20], *TCIA* https://doi.org/10.7937/k9/tcia.2018.pat12tbs (Accessed on 15 July 2020) [22], *TCIA* https://doi.org/10.7937/k9/tcia.2018.6emub5l2 (Accessed on 15 July 2020) [23], *TCIA* http://doi.org/10.7937/K9/TCIA.2016.JGNIHEP5 (Accessed on 7 May 2021) [24], *TCIA* http://doi.org/10.7937/K9/TCIA.2016.TYGKKFMQ (Accessed on 7 May 2021) [25].
